# Influence of *Oreocnide integrifolia* (Gaud.) Miq on IRS-1, Akt and Glut-4 in Fat-Fed C57BL/6J Type 2 Diabetes Mouse Model

**DOI:** 10.1093/ecam/neq014

**Published:** 2011-03-13

**Authors:** Ansarullah, Selvaraj Jayaraman, Anandwardhan A. Hardikar, A. V. Ramachandran

**Affiliations:** ^1^Department of Zoology, Faculty of Science, M. S. University of Baroda, Vadodara, India; ^2^Department of Endocrinology, Dr. ALM PGIBMS, Taramani Campus, Chennai, India; ^3^Stem Cells and Diabetes Section, National Centre for Cell Science, Pune, India

## Abstract

*Oreocnide integrifolia* (OI) leaves are used as folklore medicine by the people of northeast India to alleviate diabetic symptoms. Preliminary studies revealed hypoglycemic and hypolipidemic potentials of the aqueous leaf extract. The present study was carried out to evaluate whether the OI extract induces insulin secretion *in vivo* and *in vitro* and also whether it is mediated through the insulin-signaling pathway. The experimental set-up consisted of three groups of C57BL/6J mice strain: (i) control animals fed with standard laboratory diet, (ii) diabetic animals fed with a high-fat diet for 24 weeks and (iii) extract-supplemented animals fed with 3% OI extract along with high-fat diet for 24 weeks. OI-extract supplementation lowered adiposity and plasma glucose and insulin levels. Immunoblot analysis of IRS-1, Akt and Glut-4 protein expressions in muscles of extract-supplemented animals revealed that glucoregulation was mediated through the insulin-signaling pathway. Moreover, immunostaining of pancreas revealed increased insulin immunopositive cells in OI-extract-treated animals. In addition, the insulin secretogogue ability of the OI extract was demonstrated when challenged with high glucose concentration using isolated pancreatic islets *in vitro*. Overall, the present study demonstrates the possible mechanism of glucoregulation of OI extract suggestive of its therapeutic potential for the management of diabetes mellitus.

## 1. Introduction

India has recorded the greatest increase of diabetic patients in recent times and, with a current prevalence of 2.4% in the rural population and 11.6% in the urban population, it is estimated that by 2025 India will have the maximum number of diabetic patients [1, 2]. Although many drugs are commercially available for treating the disease, many of them are out of reach for a significant proportion of the population and are also beset with some adverse effects [3]. Treatments are essentially aimed at controlling hyperglycemia, which includes insulin (sulphonylureas, meglitinides, glucagon-like peptide (GLP) analogs, etc.) and insulin sensitizers which reduce hepatic glucose generation (metformin) or enhance peripheral glucose uptake by muscle and adipose tissue (metformin, thiazolidinediones, etc.), but no drug in vogue intrinsically exerts both the effects [4]. The use of medicinal herbs in this context is a meaningful alternative and in recognition this fact, the World Health Organization (WHO) has encouraged research in this direction and affirmed that traditional plant-based treatments for diabetes warrant further attention [5].

*Oreocnide integrifolia* (OI; Gaud.) Miq (family: Urticaceae) are shrubs/small trees mainly distributed across India, China, Bhutan, Indonesia, Laos, Myanmar, Sikkim and Thailand [6]. The roots of the *OI* plant are mixed with ginger powder and applied for treatment of rashes by the Khasi and Jayantia tribes of Meghalaya [7, 8]. They are also cooked and eaten for maintaining normal blood pressure levels by people of Manipur and an infusion prepared from the leaves is used as a decoction to alleviate diabetic symptoms [9, 10].

Type 2 diabetes mellitus is a metabolic disease with a plethora of heterogeneous interrelated manifestations and complications such as hyperglycemia, hyperinsulinemia, insulin resistance, impaired glucose tolerance and peripheral utilization, decreased hepatic glycolysis, increased gluconeogenesis, dyslipidemia, and so forth, all of which are related primarily to insulin and its action. Chronic hyperglycemia caused due to abnormalities in glucose metabolism and insulin resistance characterizes type 2 diabetes mellitus [11]. Liver being the prime center of glucose homeostasis, accumulation of hepatic lipids could contribute to insulin resistance [12]. Increased hepatic free fatty acid production and elevated plasma levels are characteristic of diabetic subjects. The higher serum free fatty acids levels lower the ability of insulin to suppress hepatic glucose production by activating gluconeogenesis and inhibiting glycolysis [13, 14]. All these metabolic alterations associated with type 2 diabetes result from a relative insufficiency of insulin to overcome peripheral insulin resistance. Different health problems such as hyperlipidemia, atherosclerosis and hypertension linked to impaired carbohydrate and lipid metabolisms are all consequences of both insulin deficiency and resistance [15, 16]. Any antidiabetic agent should exert ameliorative effect on the diabetic manifestations by enhancing insulin secretion and/or by improving/mimicking insulin action. Despite the existing treatment modalities, there is need for new treatment paradigms for type 2 diabetes mellitus to control the progressive metabolic deterioration [17]. Alternative agents being explored should be able to target the sites of basic defects ascribed to the disease, which is essentially islet dysfunction in association with insulin resistance. Molecular mechanisms underlying these basic defects also need to be evaluated to have a better understanding. To facilitate such evaluations, an appropriate clinically relevant experimental model is required. The C57BL/6J mice when fed with a high-fat diet develops all classical type 2 diabetic symptoms such as insulin resistance and insufficient islet compensation, and hence serves as the ideal model for studying pathophysiology of impaired glucose tolerance and type 2 diabetic manifestations at the molecular level [18–22]. This mouse also serves as a good model for evaluating the efficacy of new antidiabetic agents as well [23–25]. Natural products from medicinal plants are of focal interest in the discovery of new chemical entities in modern drug-discovery programs. It is in this context that we had initiated studies on OI (Gaud.) Miq used by people in northeastern part of India. Previously, we had demonstrated potent antihyperglycemic and antihyperlipidemic effects of OI leaf extract in streptozotocin-induced diabetic rats [10].

Thus, the present study was planned to evaluate whether (i) the OI extract brings about insulin secretion *in vivo* and *in vitro* and (ii) whether it is mediated through the insulin-signaling pathway.

## 2. Methods

### 2.1. Plant Extract Preparation

Fresh green leaves were collected from Imphal district (Manipur) and authenticated by the botanist, Dr Hemchand Singh, D.M. College of Science, Manipur University. A voucher specimen (#344) of the herbarium has been deposited at the department for future reference. The leaves were collected during the months of September and October, washed thoroughly and shade dried at room temperature. The dried leaves were subjected to size reduction into a coarse powder by using dry grinder and passed through a (#400) sieve. Two hundred grams of this powder was mixed with 1 l of Milli Q water (Millipore, USA) and boiled for 30 min and left to cool down to room temperature. The decoction was filtered (Whatmann #01) using a suction apparatus and the filtrate was lyophilized (Buchi, Germany) and kept in freezer at −20°C. The extractive value of the aqueous extract in terms of yield was *∼*16.9% (w/w).

### 2.2. Animals and Diets

Male C57BL/6J mice (age range: 4-5 weeks) were purchased from the National Centre for Laboratory Animal Service, National Institute of Nutrition, Hyderabad, India. To make a fully developed insulin-resistant diet-induced obese (DIO) animal phenotype, 20 animals were fed with a high-fat diet (60 kcal% fat, D12492 Research Diet, New Brunswick, NJ, USA); 20 with high-fat diet supplemented (mixed with feed) with 3% OI extract and 20 on the standard laboratory diet for 24 weeks. (BALB)/c mice (age range: 5-6 months) were used for islet culture and insulin-secretion assays. The experiment was carried out according to the guidelines of the Committee for the Purpose of Control and Supervision of Experiments on Animals, India and approved by the Animal Ethical Committee of Department of Zoology, The M. S. University of Baroda, Vadodara (Approval No. 827/ac/04/ CPCSEA).

#### 2.2.1. Metabolic Parameters

Body weight and food intake were recorded during the study period. At the end of the specified 24 weeks, animals were sacrificed, photographed for morphology of visceral adipose while muscle and pancreas were extracted for evaluation of other parameters. Hematoxylin-eosin staining was performed in paraffin sections for histological analysis of adipose tissue.

#### 2.2.2. Plasma Glucose and Insulin

Plasma glucose was measured by the tail-snipping method using One Touch Glucometer (Elegance, USA). Plasma Insulin was quantified according to manufacturer's protocol using Mouse Insulin ELISA kit (Mercodia Diagnostics, Uppsala, Sweden).

#### 2.2.3. Western Blot: Membrane Preparation

Plasma membrane and cytosolic fractions were prepared from skeletal muscles (gastrocnemius) from both control and test animals as described by Dombrowski et al. [26]. Briefly stated, 100 mg of muscle was homogenized in an ice-cold homogenization buffer (1 : 10 w/v) containing 25  mmol L^−1^ 4-(2-hydroxyethyl)-1-piperazineethanesulfonic acid (HEPES), 20 mM *β*-glycerophosphate, 2  mmol L^−1^ ethylenediaminetetraacetic acid (EDTA), 250 mmol L^−1^ sucrose, 3.3 mg L^−1^ leupeptin, 3.3 mg L^−1^ aprotinin, 100 mg L^−1^ trypsin inhibitor, 1 mmol L^−1^ PMSF, pH 7.4 using a polytron-equipped homogenizer at a precise low setting on ice. The resulting homogenate was centrifuged at 1300 × *g* for 10 min at 4°C.The supernatant was saved and pellet was resuspended in homogenization buffer and again spun at 1300 × *g* for 10 min at 4°C and the supernatant of this spin was combined with the first one and again spun at 9000 × *g* for 10 min at 4°C.The resulting supernatant was further centrifuged at 190000 × *g* for 1 h (Preparative Ultracentrifuge, Hitachi, Japan). The pellets obtained were resuspended in homogenization buffer and applied on sucrose gradients (25, 32 and 35% w/w) and centrifuged at 150000 × *g* for 16 h. Fractions at 25–32 and 32–35% interfaces were used as plasma membrane and cytosolic fractions, respectively. Protein concentration was further determined by the method of Lowry et al. [27].

#### 2.2.4. Separation of Proteins

Briefly, each sample (25 *μ*g) was subjected to heat denaturation at 96°C for 5 min with Laemmli buffer. The proteins were resolved by sodium dodecyl sulfate (SDS)-polyacrylamide gel electrophoresis (PAGE) on 10% polyacrylamide gels as described by Laemmli [28] and then electrophoretically transferred to polyvinylidene difluoride (PVDF) membrane (Amersham Biosciences, UK). The membrane was blocked with phosphate-buffered saline plus 0.3% Tween-20 (PBST) containing 10% non-fat dry milk for 2 h and then incubated with anti-GLUT-4 (1 : 2000; generous gift from Dr Samuel Cushman, NIDDK, USA), anti-IRS-1 (1 : 1000; Cell Signaling, USA), Akt-1 (1 : 1000; Cell Signaling, USA) and *β*-actin (1 : 2000; Santa Cruz Biotechnology, USA) primary antibodies overnight. After three washes with PBST, the membrane was re-blocked and incubated with secondary antibody (horseradish peroxidase-conjugated donkey anti-rabbit IgG; 1 : 5000; Sigma, St. Louis, MO, USA) for 2 h at room temperature. The blots were then rinsed in Tris-buffered saline with 0.05% Tween 20 and immunoreactive bands were detected by the Enhanced Chemiluminescence Reagents (ECL; Amersham Biosciences, UK). Images were captured with a ChemiDoc XRS system (Bio-Rad Laboratories, CA, USA). Later, the membranes were incubated in stripping buffer (50 mL containing 62.5 mmol L^−1^ Tris-HCl (pH: 6.8), 1 g SDS and 0.34 mL *β*-mercaptoethanol) at 55°C for 40 min. After this, the membrane was reprobed using a *β*-actin antibody (1 : 2000). All protein bands were quantified using (Image J software, NIH, USA) and normalized against internal control *β*-actin.

#### 2.2.5. Immunostaining and Confocal Microscopy

The pancreas were aseptically removed from the respective treatment groups and fixed in 4% fresh paraformaldehyde. The tissues were subsequently embedded in paraffin wax and sectioned at 5 *μ*m thickness with a microtome (Leica, Wetzlar, Germany) and mounted on poly-l-lysine (Sigma) coated slides. Slides were de-paraffinized, downgraded in xylene and alcohol and blocked with 4% normal donkey serum and then incubated with antisera. Guinea pig anti-insulin antibody (Linco Research Inc, St. Charles, MO, USA), mouse antiglucagon (Sigma), were used at 1 : 100 dilutions. Alexa-Fluor 488 and Alexa-Fluor 546 F (ab′)_2_ secondary antibodies (Molecular Probes, OR, USA) were used at 1 : 200 dilution. Hoechst 33342 was used to visualize nuclei. Primary antibodies were incubated overnight at 4°C, washed with calcium-magnesium-containing PBS and then incubated with the secondary antibodies at 37°C for 1 h. Slides were washed extensively in PBS and mounted in Vectasheild (Vectorlabs). Confocal images were captured using a Zeiss LSM 510 laser scanning microscope using a 63 × 1.3 oil objective with optical slices *∼*0.8 *μ*m. Magnification, laser and detector gains were set below saturation and were identical across samples.

#### 2.2.6. Isolation of Islets

Islet isolation was performed according to the method of Lacy and Kostianovsky, Shewade et al. [29, 30]. Groups of three BALB/c mice were killed by cervical dislocation, and splenic pancreas was removed under sterile conditions without ductal injection and distention. Briefly, the pancreas was cut into small pieces/chopped finely *∼*1 mm^2^ and was subjected to enzymatic digestion for 10–12 min by mechanical vigorous shaking in water bath maintained at 37°C. The dissociation medium consisted of Dulbecco's Modified Minimum Essential Medium (DMEM) supplemented with Collagenase type V (1 mg mL^−1^; Sigma), and 2% BSA fraction V (Sigma). The tissue digested was then centrifuged at 1500 × *g* for 10 min, washed twice in PBS (pH: 7.4) and seeded in culture flasks (25 cm^2^; Nunc, Denmark) containing RPMI-1640 (Hyclone, USA) supplemented with 10%(v/v) FBS (Hi-Media, India), 100 U mL^−1^ penicillin and 100 U mL^−1^ streptomycin under 95% O_2_ and 5% CO_2_ atmosphere at 37°C (Thermo, USA) in air. Under these culture conditions, most of the acinar cells degenerate within 48 h leaving islets. After 48 h of incubation, islets were separated from exocrine pancreas by hand-picking using a binocular stereomicroscope. The Islet specificity was assessed using dithizone (Hi-Media, Mumbai, Maharashtra, India) and Trypan blue staining was performed for viability.

#### 2.2.7. Insulin Secretion Assay

Isolated islets were cultured at 37°C in a humidified atmosphere of 5% CO_2_ in air in RPMI-1640 medium containing 11.1 mM glucose, 10% FBS and antibiotics. Islets were seeded at a concentration of 100 islets per well in 24-well plates (Falcon, NJ, USA) and allowed to attach overnight prior to acute tests. Wells were washed 3 times with Krebs-Ringer bicarbonate buffer (KRB; 115 mM sodium chloride (NaCl), 4.7 mM potassium chloride (KCl); 1.3 mM calcium chloride (CaCl_2_), 1.2 mM potassium dihydrogen phosphate (KH_2_PO_4_), 1.2 mM magnesium sulfate (MgSO_4_), 24 mM sodium bicarbonate (NaHCO_3_), 10 mM HEPES, 1 g L^−1^ BSA, 1.1 mM glucose; pH: 7.4) and preincubated for 1 h at 37°C. Unless otherwise stated, wells were then incubated for 1 h with 1 mL KRB at 4.5 mM and 16.7  mM glucose, and OI extract (10, 50, 100 and 250 *μ*g mL^−1^). Aliquots were removed from each well, centrifuged (1500× g for 5 min, at 4°C), and assayed for insulin using mouse insulin ELISA kit and protein concentration was determined.

### 2.3. Statistical Analysis

Statistical evaluation of the data was done by one-way Analysis of Variance (ANOVA) followed by Bonferroni Multiple comparison test. The results are expressed as mean ± SEM using Graph Pad Prism version 3.0 for Windows (Graph Pad Software, San Diego, CA, USA).

## 3. Results

### 3.1. Metabolic Parameters

**Figure 1:**
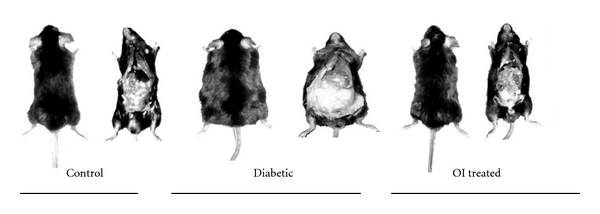
Control, diabetic and OI extract-treated mice opened to expose the viscera to show the variation in visceral adiposity.

**Figure 2:**
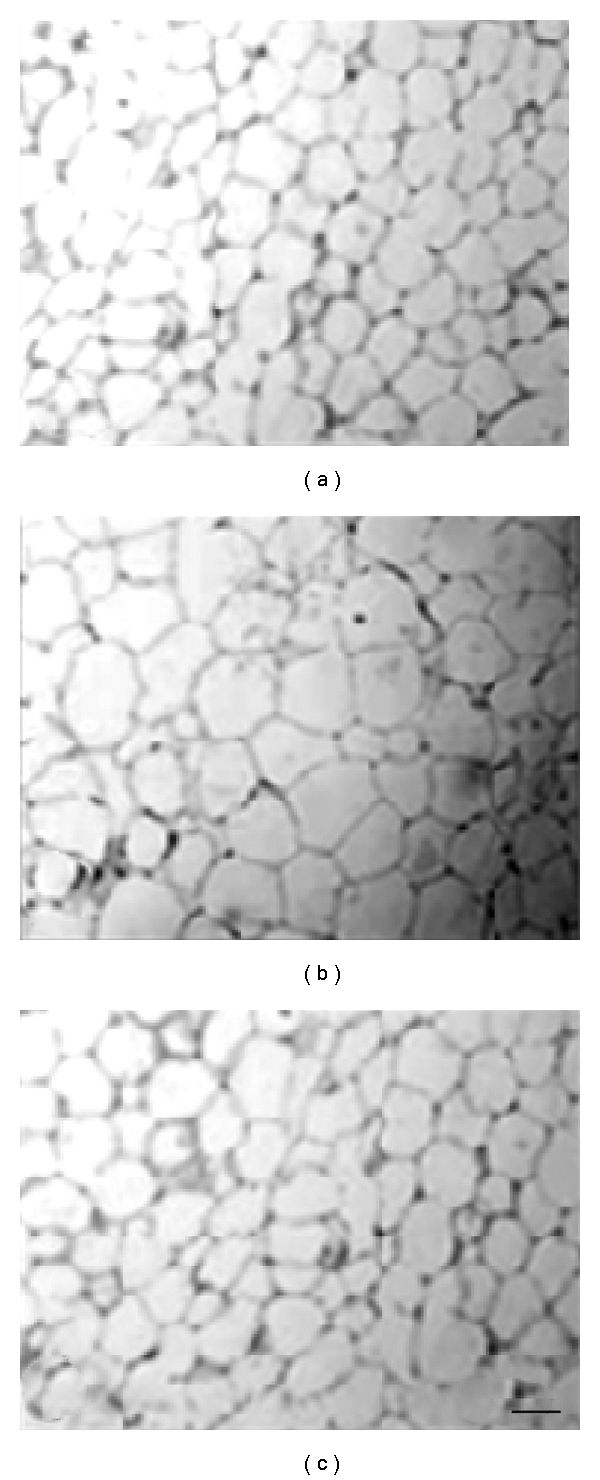
Histomicrograph of sections of adipose tissue from control (a), diabetic (b) and OI extract-treated (c) mice to depict difference in adipocyte dimensions. Scale bar represents 100 *μ*m. Magnification 450×.

The body weight of diabetic animals increased significantly up to 24 weeks on high-fat diet by 43% as compared to the control mice. Simultaneous supplementation with OI extract reduced bodyweight gain to only about 13% which was confirmable by morphological observation of decreased visceral adiposity (Figure 1). Histological analysis of epididymal fat pads revealed increased adipocyte diameter in diabetic mice which was effectively checked by OI extract supplementation (Figure 2). Food consumption decreased significantly by 28% in diabetic mice as compared to controls whereas OI extract fed diabetic mice did not show any significant change (Table 1).

### 3.2. Plasma Glucose Levels and Insulin Titer

Plasma blood glucose level was increased by 64% in high-fat diet mice compared to controls (5.70 ± 0.71 versus 16.12 ± 0.81 mmol L^−1^) at the end of 24 weeks (Table 2). OI extract supplementation along with high-fat diet resulted in only 20% increase in plasma glucose levels. The plasma insulin titer decreased in high-fat diet-fed diabetic mice, while simultaneous supplementation with OI extract showed a non-significant change.

#### 3.2.1. Glucose-Induced Insulin Secretion

Islets isolated from BALB/c mice were incubated with various concentrations (10, 50, 100 and 250 *μ*g mL^−1^) of OI extract along with basal (4.5 mM) and stimulated (16.7 mM) glucose concentrations (Table 3). There was a dose-dependent effect with 100 and 250 *μ*g mL^−1^ showing maximal insulin secretion at the end of 60 min of incubation.

#### 3.2.2. Western Blot Analysis

**Figure 3:**
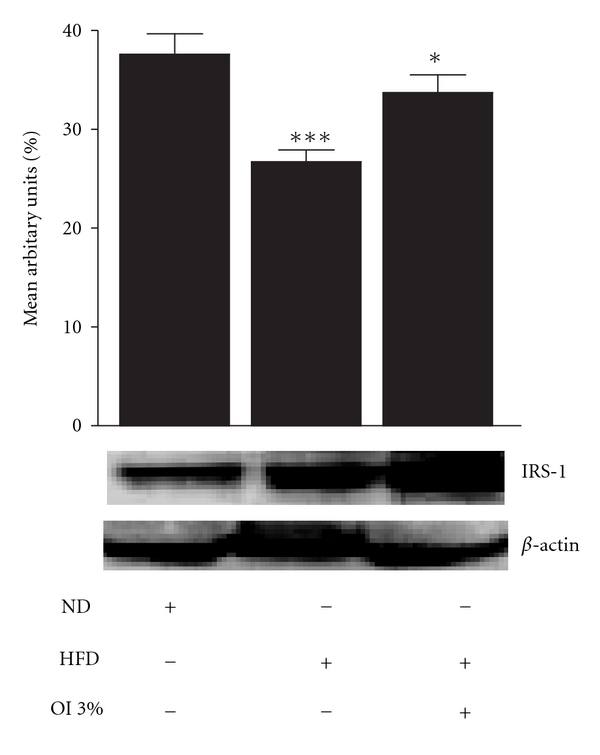
IRS-1 protein expression in skeletal muscles of control and experimental animals. Mean ± SEM of six animals. ***P < .001, *P < .05; where * = control versus diabetic, control versus OI extract-treated.

**Figure 4:**
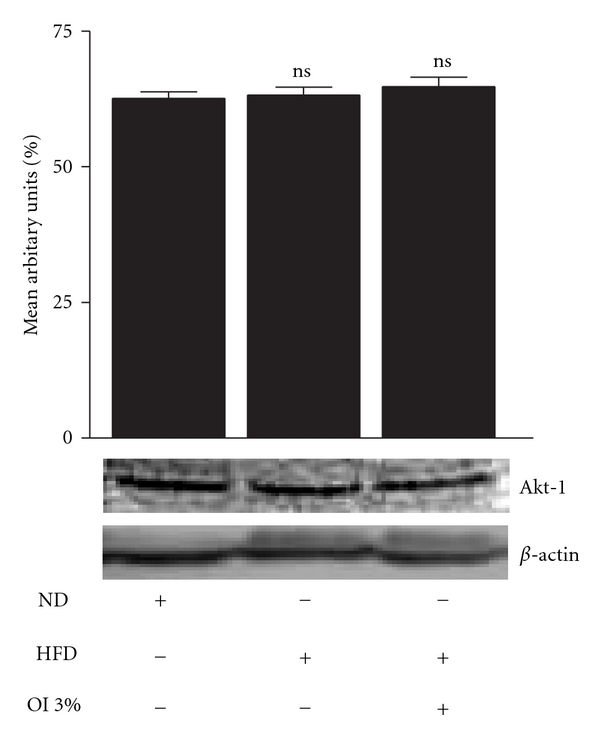
Akt-1 protein expression in skeletal muscles of control and experimental animals. Mean ± SEM of six animals ns = *P* > .05.

**Figure 5:**
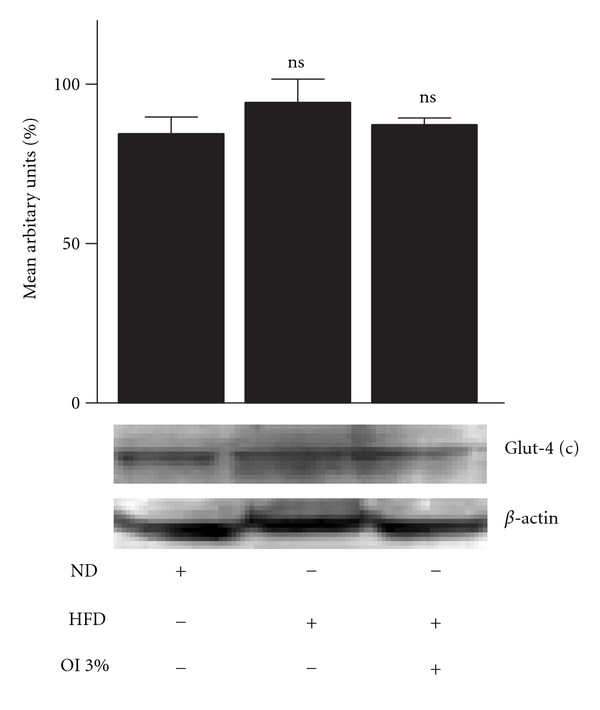
Glut-4 cytosolic protein expression in skeletal muscles of control and experimental animals. Mean ± SEM of six animals ns = *P* > .05.

**Figure 6:**
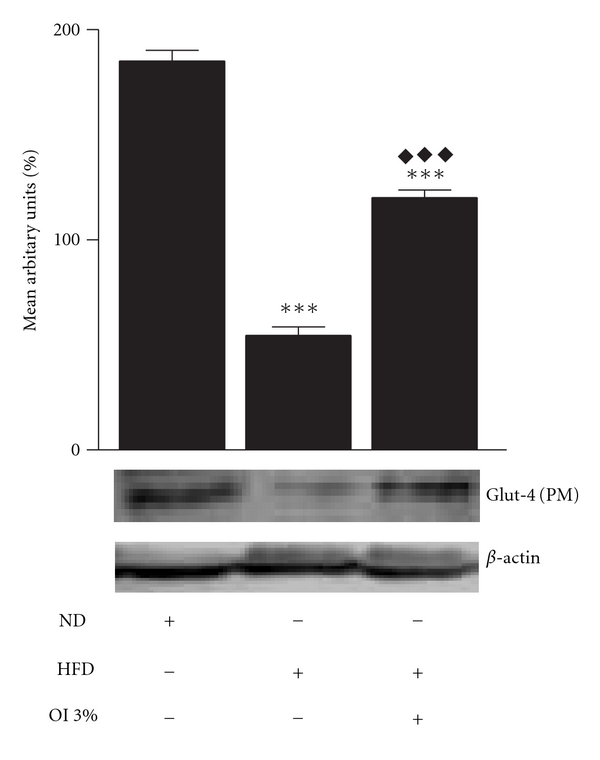
Glut-4 plasma membrane protein expression in skeletal muscles of control and experimental animals. Mean ± SEM of six animals. ^∗∗∗/♦♦♦^ *P* < .001; where * = control versus diabetic, control versus OI extract-treated and ♦ = diabetic versus OI extract-treated.

Immunoblot analysis was carried out in control, diabetic and OI extract-fed groups wherein protein-expression pattern of molecules involved in the insulin-signaling pathway was evaluated in the gastrocnemius muscle. IRS-1 expression in muscle (cytosolic fraction) was decreased significantly (*P* < .01) in diabetic mice compared with controls while OI extract-supplemented animals depicted higher near-normal level when compared with diabetic animals (Figure 3). However, AKT-1 expression (cytosolic fraction) of control, diabetic or OI-treated group did not show any significant difference (Figure 4). Effect on Glut-4 distribution was evaluated in both cytosolic and plasma membrane fractions (Figures 5 and 6). Cytosolic fraction showed no significant changes in Glut-4 protein expression in either control or experimental groups of animals. However, membrane glut-4 expression was remarkably reduced (*P* < .001) in diabetic animals and there was a conspicuous maintenance of Glut-4 expression in OI-supplemented group although still lesser than in control animals.

#### 3.2.3. Insulin/Glucagon Immunostaining of Pancreas

**Figure 7:**
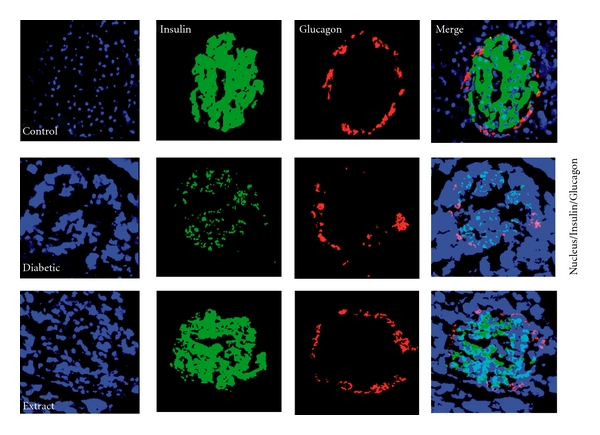
Guinea pig anti-insulin antibody (Linco Research Inc, MO) and mouse antiglucagon (Sigma) antibodies were used at 1 : 100 dilutions. Alexa Flour-488 and Alexa Flour 546 F(ab)_2_ secondary antibodies (Molecular Probes) were used at 1 : 200 dilution. Hoecsht 33342 was used to visualize nuclei. Confocal images were captured using a Zeiss LSM 510 laser scanning microscope using a ×63/1.3 oil objective with optical slices *∼*0.8 *μ*m.

Pancreas from different groups of animals were immunostained for insulin at the end of the experimental period (Figure 7). Diabetic mice showed weak staining for insulin while the OI extract-supplemented group showed remarkable insulin immunopositivity closest to the control islet response. However, there was no significant change in glucagon-positive cells in control and experimental groups.

## 4. Discussion

**Figure 8:**
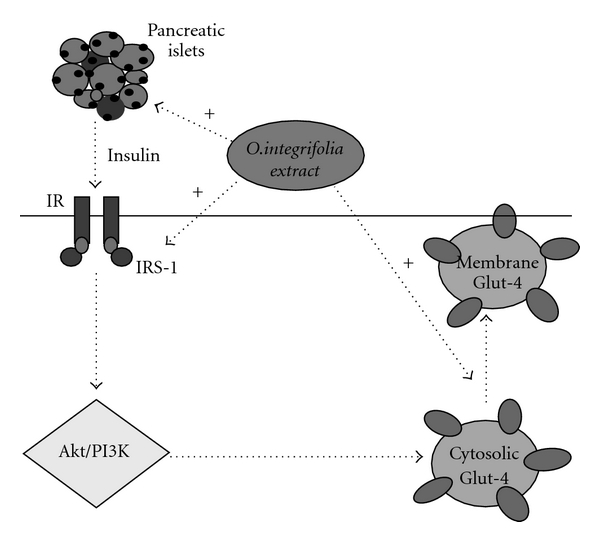
The figure summaries varied actions of OI extract (dark dotted arrows with +sign) on Glut-4 translocation from cytosol to plasma membrane through activation of IRS-1 in skeletal muscles. In addition, OI extract demonstrated increased glucose-induced insulin secretion in isolated islets (*in vitro*) and increased insulin-immunopositive cells in pancreas (*in vivo*).

The high-fat diet-fed C57BL/6J mouse is an ideal model for studying mechanisms of impaired glucose tolerance along with insulin resistance leading to type 2 diabetes marked by islet dysfunction and for developing novel therapeutic interventions [31]. The study has tried to evaluate the influence of OI extract on glucose-uptake mechanisms in the muscle of diabetic and non-diabetic C57BL/6J mouse and also the efficacy of extract in inducing insulin secretion from isolated islets from BALB/c mice. The C57BL/6J mouse developed type 2 diabetic manifestation when fed continuously for 24 weeks with a high-fat diet. Diabetic induction was marked by loss of insulin-positive cells in islets, >200% decrease in fasting plasma glucose and >20% decrease in plasma insulin level accompanied by decreased expressions of IRS-1 and membrane Glut-4 in the muscle. Supplementation with OI extract is able to ameliorate the diabetic manifestations marked by noticeable insulin immunoreactivity in the islets and improved plasma insulin titer and glycemic level suggesting, both an insulinogenic action as well as insulin sensitivity-potentiating effect of the OI extract. In recent times, herbal extracts and compounds isolated from plants have been shown to have various effects on pancreas, such as *β*-cell proliferation, insulin synthesis and secretion, suggesting the potential role of medicinal herbs in combating insulin resistance and insufficiency associated with diabetes; further, Huo et al. [32] showed increased serum insulin levels on treatment with a powder mixture of eight herbal components. Qin et al. [33] showed that *Gosha-jinki-gan* (a herbal complex) ameliorates abnormal insulin signaling. Similarly, Muniappa et al. [34] showed insulin secretogogue activity and cytoprotective role of *Scorparia dulcis* (Sweet Broomweed) and Jayaprakasan et al. [16] demonstrated the ability of anthocyanins and ursolic acid isolated from the Cornelian cherry (*Cornus mas*) for enhanced islet function and elevated circulating serum insulin levels. Similarly, Leu et al. [35] have also reported competence of an extract of *Angelica hirsituflora* to serve as an insulin secretogogue. The OI extract in our present study seems capable of acting as a secretogogue as well, and this is confirmed by the *in vitro* ability of the extract to bring about insulin release from isolated BALB/c mice islets in a dose-dependent manner. All doses of OI extract-induced 80%–100% proportionate increase in insulin secretion when the glucose concentration in the medium was increased from 4.5 to 16.7 mM. Taken with the herein noted effect on pancreatic islets, the current protective effects seen suggest potential of OI extract to act at the levels of both insulin production as well as insulin action. The principal action of insulin on glucoregulation is through peripheral tissue uptake and metabolism. Muscle and adipose tissue are the main insulin-sensitive tissues involved in this process. A defect in insulin receptor or post-receptor signalling mechanism can have profound effect in terms of insulin insensitivity. Number of molecular lesions in muscle and adipose tissue are known to exacerbate diabetic symptoms and can even be a major cause of type 2 diabetes [3]. Western blot analysis of some proteins involved in insulin-induced glucose metabolism in the muscle tissue in the present study has revealed significantly decreased membrane Glut-4 and cytosolic IRS-1 expressions in high-fat diet-induced type 2 diabetes. However, Akt-1 was not significantly compromised. Apparently, high-fat diet-induced type 2 diabetes in C57BL/6J mice has defective peripheral glucose utilization due to deficiency in IRS-1, resulting in downstream defect in Akt-1-PI-3 kinase activation. This in turn leads to non-phosphorylation of Glut-4 and its membrane translocation contributing to reduced glucose transport [36]. Glut-4 is the insulin-sensitive glucose transporter in skeletal muscle and adipose tissue and its importance in whole body glucose metabolism has been elucidated using experimental models of Glut-4 null mice [37]. Recently, Liu et al. [38] demonstrated the action of *Dang Gui Bu Xue Tang* (herbal formulation) on Glut-4 translocation in fructose-fed rats. Of late, attention has shifted to herbal extracts/constituents as potential agents for treatment of type 2 diabetes. Identifying targets of action of herbal preparations is an essential aspect of therapeutic drug development in combating molecular lesions characteristic of insulin resistance and type 2 diabetes. There is, therefore, a need to evaluate the efficacy of herbal extracts/principles on a composite scale with regard to molecular defects in peripheral insulin resistance/glucose utilization. Although there are a few studies on plant products/principles on this aspect, their obvious drawbacks lie in the fact that these studies are either on cultured myoblast cells or on alloxan/streptozotocin-induced type 1 diabetic animals and that too restricted to only Glut-4 concentration or glucose uptake [39–43]. The present study, in this context, has tried to assess cytosolic and membrane Glut-4, Akt-1 and IRS-1 expressions. The study clearly shows that OI extract is able to prevent, high-fat diet-induced type 2 diabetic manifestations of under-expression of IRS-1 protein and hampered membrane translocation of Glut-4 transporter in the skeletal muscle (Figure 8). Overall, the present study effectively shows that diet-induced type 2 diabetes is characterized by significant islet dysfunction (*β*-cell loss), with consequent hypoinsulinemia and peripheral deficiency in glucose uptake by insulin-sensitive peripheral tissues (muscle) and that, OI extract is adequately competent to counteract these effects of diet-induced diabetic manifestations. Further, we have also recorded decreased hepatic ^14^C glucose oxidation and increased glucogenesis coupled with gluconeogenesis as marked by tissue glycogen content and mRNA levels of glucose-6-phosphatase, glucokinase and phosphoenolpyruvate carboxykinase (Ansarullah, Unpublished). Our previous studies have recorded dose-dependent hypoglycemic and hypolipidemic effects of OI extract in streptozotocin-induced diabetic rats [10]. Moreover, preliminary assay of phytoconstituents present in OI extract has revealed the presence of sterols, saponins, terpenoids, flavonoid glycosides and sugars. (Ansarullah, personal communication). Having demonstrated the potent effect of OI extract in combating type 2 diabetic manifestations, our further focus is on isolation of principles/compounds from the extract to test the bioactive molecule(s) for development as an alternative therapeutic agent against type 2 diabetes.

## Conflict of Interest

None declared.

## Figures and Tables

**Table 1:** Body weight and food intake.

	Control	Diabetic	OI extract treated
Body weight initial (g)	24.2 ± 0.23	23.7 ± 0.41^ns^	22.6 ± 0.78^ns/ns^
Body weight final (g)	26.1 ± 0.43	41.3 ± 1.81***	29.3 ± 0.48^ns/♦♦♦^
Weight gain (g)	1.9 ± 0.37	17.6 ± 0.67***	6.7 ± 0.32^∗∗∗/♦♦♦^
Food intake (g day^−1^ per mouse)	2.6 ± 0.17	1.87 ± 0.13*	2.2 ± 0.18^ns/ns^

Effect of OI extract on body weight and food intake in control and experimental animals. Mean ± SEM of six animals.

^∗∗∗/♦♦♦^ *P* < .001, *P < .05, ns = *P* > .05; where * = control versus diabetic, control versus OI extract-treated and ♦ = diabetic versus OI extract-treated.

**Table 2:** Glucose and insulin.

	Control	Diabetic	OI extract-treated
Glucose (mmol L^−1^)	5.70 ± 0.71	16.12 ± 0.81***	8.90 ± 0.56^∗/♦♦♦^
Insulin (pmol L^−1^)	88.32 ± 7.27	66.39 ± 6.63^∗/ns^	72.43 ± 10.25^ns/ns^

Effect of OI extract on plasma levels of glucose and insulin in control and experimental animals. Mean ± SEM of six animals.

^∗∗∗/♦♦♦^ *P* < .001, *P < .05, ns = *P* > .05; where * = control versus diabetic, control versus OI extract-treated and ♦ = diabetic versus OI extract-treated.

**Table 3:** Glucose-induced insulin secretion.

	4.5 mM Glucose	16.7 mM Glucose
	pmol l^−1^ per 100 islets	pmol l^−1^ per 100 islets
Control	50.0 ± 1.66	87.5 ± 3.31
10 *μ*g mL^−1^ OI extract	47.7 ± 1.94	101.3 ± 1.31**
50 *μ*g mL^−1^ OI extract	61.3 ± 2.15*	112.4 ± 1.80**
100 *μ*g mL^−1^ OI extract	68.4 ± 1.52**	125.6 ± 1.30**
250 *μ*g mL^−1^ OI extract	67.0 ± 2.10*	142.9 ± 0.83**

Effect of OI extract on basal and stimulated levels of glucose induced insulin secretion. Mean ± SEM of six animals.

**P < .01, *P < .05; where * = control versus diabetic, control versus OI extract-treated. Mean ± SEM of five independent experiments. *P < .01 when compared to respective controls and **P < .01 when compared to respective controls.
